# Hydrodynamic Behavior of Self-Propelled Particles in a Simple Shear Flow

**DOI:** 10.3390/e24070854

**Published:** 2022-06-22

**Authors:** Tingting Qi, Jianzhong Lin, Zhenyu Ouyang

**Affiliations:** 1 State Key Laboratory of Fluid Power Transmission and Control, Zhejiang University, Hangzhou 310027, China; 22024006@zju.edu.cn (T.Q.); ouyangzhenyu@zju.edu.cn (Z.O.); 2 Laboratory of Impact and Safety Engineering of Ministry of Education, Ningbo University, Ningbo 315201, China

**Keywords:** self-propelled particles, hydrodynamic properties, simple shear flow, immersed boundary-lattice Boltzmann method

## Abstract

The hydrodynamic properties of a squirmer type of self-propelled particle in a simple shear flow are investigated using the immersed boundary-lattice Boltzmann method in the range of swimming Reynolds number 0.05 ≤ *Re_s_* ≤ 2.0, flow Reynolds number 40 ≤ *Re_p_* ≤ 160, blocking rate 0.2 ≤ *κ* ≤ 0.5. Some results are validated by comparing with available other results. The effects of *Re_s_*, *Re_p_* and *κ* on the hydrodynamic properties of squirmer are discussed. The results show that there exist four distinct motion modes for the squirmer, i.e., horizontal mode, attractive oscillation mode, oscillation mode, and chaotic mode. Increasing *Re_s_* causes the motion mode of the squirmer to change from a constant tumbling near the centerline to a stable horizontal mode, even an oscillatory or appealing oscillatory mode near the wall. Increasing the swimming intensity of squirmer under the definite *Re_s_* will induce the squirmer to make periodic and stable motion at a specific distance from the wall. Increasing *Re_p_* will cause the squirmer to change from a stable swimming state to a spiral motion or continuous rotation. Increasing *κ* will strengthen the wall’s attraction to the squirmer. Increasing swimming intensity of squirmer will modify the strength and direction of the wall’s attraction to the squirmer if *κ* remains constant.

## 1. Introduction

Various movements of self-propelled particles play an essential role in the medicinal, biophysical and engineering applications. Sperm, bacteria, protists and algae are examples of self-propelled microorganisms in nature. They achieve self-propulsion by using their own motor organs such as cilia and flagella, tail and fins, cell deformation and so on. Movement of microorganisms is associated with a variety of biological activities such as sperm swimming in mammalian cervical mucus [1], biofilm formation [2], paramecia swimming to avoid predators [3], and bacteria and algae coordinating their movement to nutrient-rich habitats [4]. The motion of self-propelled particles in the flow will be affected by the fluid motion, with the motion of self-propelled particles in the shear flow being of special importance [5,6,7].

Alqarni and Bearon [8] found that cells would generate a spiral swimming trajectory in the weak shear flow but could achieve a stable equilibrium direction in the strong shear flow. They also numerically simulated the trajectories of cells in a non-uniformly sheared vertical channel flow and found that helical swimming cells would aggregate toward or away from the channel center. Ishimoto and Crowdy [9] provided an analytical solution for the motion of circular self-driven particles in a simple shear flow near a non-slip wall, and demonstrated that particles couldn’t migrate stably at a fixed distance from the wall, but could only oscillate periodically along the wall or move away from it. According to the results given by Ishimoto and Gaffney [10], the fluid rheology could be used to direct sperm into the egg, and sperm moved under the combined effect of self-driving, wall constraint and fluid shear force. Jiang and Chen [11] investigated the dispersion model of dilute suspensions of self-propelled particle in a confined flow and found that the accumulation of spherical particles in shear flow would reduce overall dispersion, whereas the accumulation of rod-like self-propelled particles in shear flow would increase dispersibility because the particles were aligned with the streamlines. Brady et al. [12] simulated the stress tensor and diffusion tensor of spherical particles in the simple shear flow and pressure-driven flow. Hagen et al. [13] studied the Brownian motion of self-propelled particles in a linear shear flow, and indicated that the particles moved at a constant speed along the wave direction and were subjected to a constant torque. In addition, Wagner and Kalman [14] developed the flow-ultra-small-angle neutron scattering method for probing colloidal microstructures under steady-state flow conditions, and found that the formation of water clusters caused reversible shear thickening in colloidal suspensions due to the predominance of short-range lubrication- hydrodynamic interactions at relatively high shear rates. Siebenbürger et al. [15] conducted comprehensive research of viscoelasticity and shear flow of concentrated amorphous colloidal suspensions. Lettinga and Dhont [16] investigated the phase and flow behavior of rod-shaped particles in the shear flow, and calculated the whole phase diagram of rod-shaped particles from low concentration to two-phase area and to nematic region. Blaak et al. [17] investigated the effect of shear flow on homogeneous crystal nucleation and found that a uniform shear rate could significantly reduce crystal nucleation rate while increasing critical nucleation size. They also indicated that the nuclei orientation was inclined with respect to the shearing direction. Dhont and Nagele [18] examined the critical viscoelastic behavior of colloidal suspensions and found that the microstructural distortion generated by static shear flow had a significant impact on the spectrum of the linear viscoelastic response function.

It can be seen from the above research that there is still a lack of studies on the effects of swimming Reynolds number, flow Reynolds number and blocking rate on the hydrodynamic properties and stable equilibrium position of self-propelled particle. Therefore, the aim of this study is to numerically simulate the hydrodynamic properties of self-propelled particles moving in a simple shear flow using the lattice Boltzmann-immersed boundary method, and explore the effects of swimming Reynolds number, flow Reynolds number and blocking rate on the hydrodynamic properties and stable equilibrium position of self-propelled particle.

## 2. Basic Model

### 2.1. Squirmer Model

The squirmer model proposed by Lighthill [19] and Blake [20] has been widely used in the study of self-propelled particles. The model of two-dimensional squirmer driven with tangential surface velocity is:

(1) $$u_{\theta }=B_{1}\sin\theta +2B_{2}\sin\theta \cos\theta ,$$

the squirmer’s self-driving velocity is determined by the first term on the right hand side of Equation (1), $U{/}_{Re=0}=B_{1}/2$ and an irrotational velocity field with a decay rate of $1/r^{2}$ is generated; the second term is related to the squirmer’s stress, which causes the Stokes flow to decay at a rate of 1/r, generating vortices near the squirmer surface [21]. Squirmers are classified into three categories based on the values of $\beta =B_{2}/B_{1}(B_{1}>0)$: puller (β>0), pusher (β<0), and neutral squirmer (β<0). Puller, such as Chlamydomonas, creates thrust from the front with a breaststroke-like motion. Pusher, such as *E. coli*, pushes itself forward with their backward flagella [22].

Squirmer is assumed a rigid body, and the squirmer’s motion is described by the Newton’s second law:

(2) $$m\frac{d^{2}x_{c}}{dt^{2}}=F,\;\frac{d\left(J\cdot \Omega \right)}{dt}=T\;,$$

where m and $x_{c}$ represent the squirmer’s mass and centroid position, respectively; J and Ω represent the squirmer’s moment of inertia and angular velocity, respectively; F and T represent the force and torque exerted by the fluid on the squirmer, respectively.

### 2.2. Collision Mode

There will be an interaction between the squirmer and wall when the squirmer is close to the wall. The short-range repulsion model provided by Glowinski et al. [23] is employed to avoid the overlapping of squirmer and the wall:

(3) $$f_{r}=\{\begin{array}{c} \frac{C_{m}}{\varepsilon }\left(\frac{d-d_{min}-∆r}{∆r}\right)e_{r},\;d\leq d_{min}+∆r\\ \left(0,0\right),\;d>d_{min}+∆r \end{array},$$

where $C_{m}=MU^{2}/a_{0}$ is the characteristic force; M,U are $a_{0}$ the squirmers’ mass, velocity and radius, respectively; $\varepsilon =10^{-4}$ is a constant positive value; d is the distance between the squirmer and the wall; $d_{min}=a_{0}$ is the minimum possible distance between the squirmer and the wall; ∆r=2∆x represents the size of the two lattices in the numerical simulation, which is the area where the repulsion exists; $e_{r}$ indicates that the center of the squirmer points to the normal direction of the wall.

## 3. Numerical Methods and Verification

### 3.1. Immersion Boundary-Lattice Boltzmann Method

The immersed boundary-lattice Boltzmann method [24,25] is utilized. In this method, the regular Euler grid is used in the flow and the lattice Boltzmann equation is solved with the velocity discrete model of DdQm to obtain the macroscopic information of the flow. The Lagrangian grid is used to model particles moving in the flow, and two sets of grids are used to exchange force and velocity information between the Lagrangian points of the particle border and the Euler points of the surrounding flow.

The N-S equation for an incompressible flow is:

(4) $$\frac{\partial u}{\partial t}+\left(u\cdot \nabla \right)u=-\frac{\nabla p}{\rho }+\frac{\mu }{\rho }\nabla^{2}u+f$$

(5) ∇·u=0  ,

where ρ, u  and p are the fluid density, velocity and pressure, respectively; f is the external force exerting on the fluid. 

The D2Q9 velocity model [26] is employed and the appropriate velocity vector is:

(6) $$e_{\alpha }=\{\begin{array}{c} \left(0,0\right)\;\alpha =0\;\\ \left(\pm 1,0\right),\left(0,\pm 1\right)\;\alpha =1\sim 4\\ \left(\pm 1,\pm 1\right)\;\alpha =5\sim 8 \end{array}\;.$$

The corresponding single relaxed lattice Boltzmann equation with external force term is:

(7) $$f_{\alpha }\left(x+e_{\alpha }∆t,t+∆t\right)=f_{\alpha }\left(x,t\right)-\frac{1}{\tau }\left[f_{\alpha }\left(x,t\right)-f_{\alpha }^{eq}\left(x,t\right)\right]+∆t\frac{w_{\alpha }\rho }{c_{s}^{2}}e_{\alpha }\cdot f,$$

where ∆t is the time step of simulation; τ is the relaxation time; $\;f_{\alpha }\left(x,t\right)$ is the density distribution function of fluid particle for the velocity direction $e_{\alpha \;}$ in x at time t; $c_{s}=c/\sqrt{3}=1/\sqrt{3}$ is the speed of sound; f is an external force; $w_{\alpha }$ is the weight function, $w_{0}=4/9,\;w_{\alpha }=1/9\;\mathrm{for}\;\alpha =1-4,\;w_{\alpha }=1/36\;\mathrm{for}\;\alpha =5-8;\;f_{\alpha }^{eq}$ is the equilibrium distribution function:

(8) $$f_{\alpha }^{eq}\left(x,t\right)=\rho w_{\alpha }\left[1+\frac{e_{\alpha }\cdot u}{c_{s}^{2}}+\frac{{\left(e_{\alpha }\cdot u\right)}^{2}}{2c_{s}^{2}}-\frac{u^{2}}{2c_{s}^{2}}\right].$$

The macroscopic velocity and density of the fluid are:

(9) $$\rho =\sum f_{\alpha }\;,\;\rho u=\sum f_{\alpha }e_{\alpha }\;.$$

**Figure 1:**
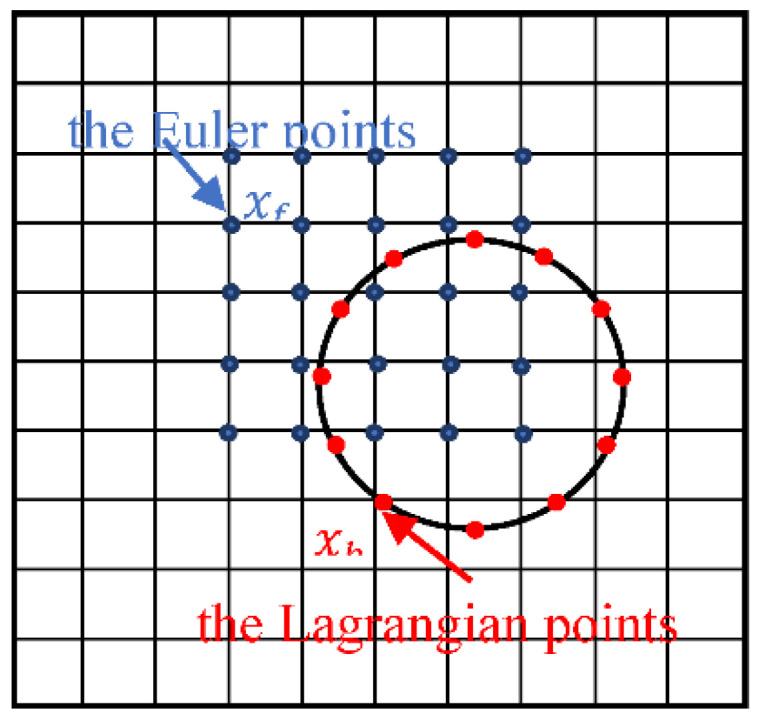
Distribution of Euler points and Lagrangian points.

For the exchange of velocity and force information between the solid boundary and the flow, the force exerted on the solid boundary by the fluid is:

(10) $$F\left(x,t\right)=\frac{U^{d}\left(x,t+∆t\right)-U^{*}\left(x,t+∆t\right)}{∆t}\;,$$

where $U^{d}\left(x,t\right)$ is determined by the motion of the particle. As shown in Figure 1, at the point $x_{b}$, $U^{d}\left(x_{b},t\right)$ is the sum of translational and rotational velocities of the particle,$U^{*}\left(x_{b},t\right)$ is obtained by interpolating the fluid around the boundary:

(11) $$U^{*}\left(x_{b},t\right)=\sum_{f}D\left(x_{f}-x_{b}\right)\cdot u^{*}\left(x_{f},t\right),$$

where $u^{*}\left(x_{f},t\right)$ is the fluid velocity at $x_{f}$ without considering the external force; D(x) is a two-dimensional Dirac delta function [27].

Similar to Equation (10), the forces exerted on the fluid by the solid boundary is:

(12) $$f\left(X_{f},t\right)=\sum_{b}D\left(X_{f}-X_{b}\right)\cdot F\left(X_{b},t\right).$$

where *F* is the force exerted on the solid boundary by fluid, *D* is the Dirac delta function.

### 3.2. Verification of Numerical Method

**Figure 2:**
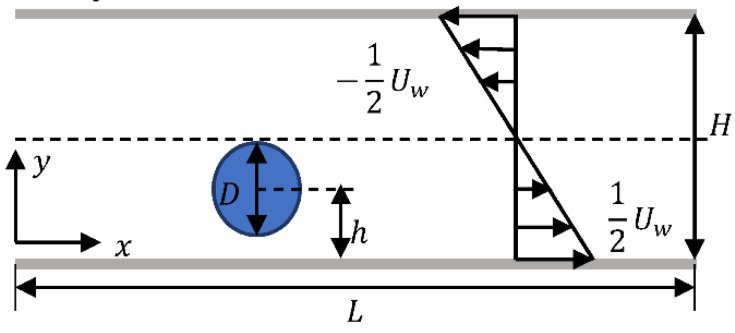
Particle moving in a simple shear flow.

**Figure 3:**
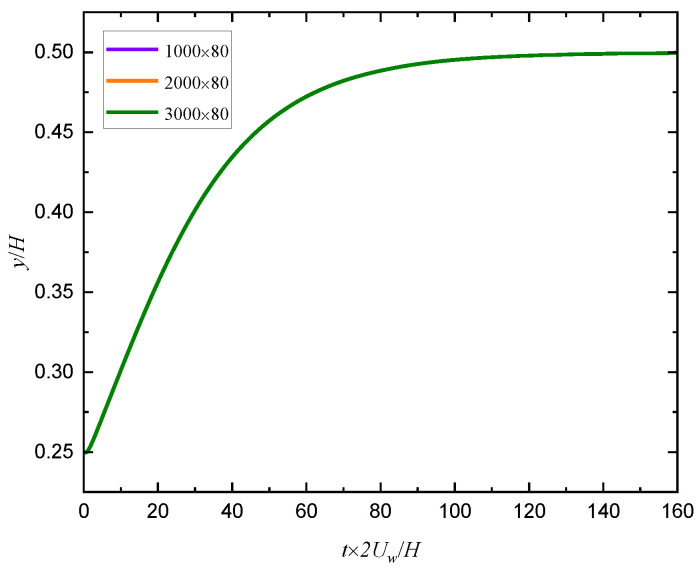
Particle trajectories for different channel lengths.

**Figure 4:**
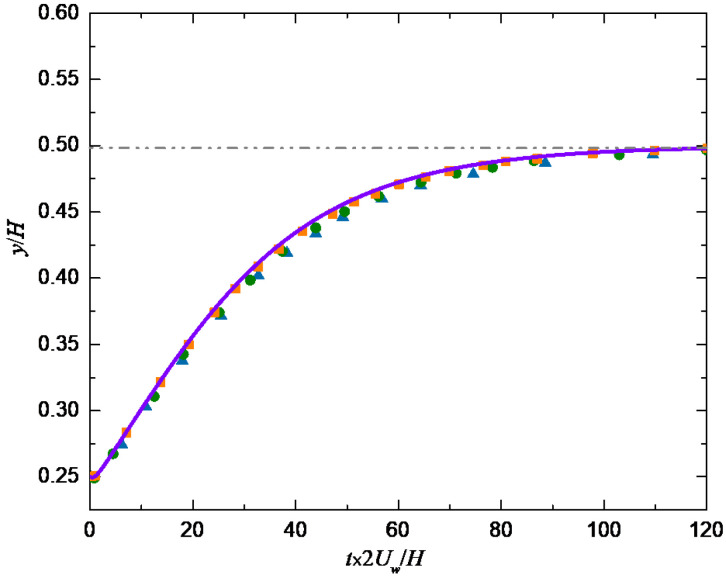
Comparison of particle trajectories. 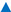: Ref. [28]; 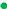: Ref. [29]; 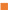: Ref. [30]; —: present result.

As shown in Figure 2, the motion of a single particle in a Newtonian shear flow is simulated to verify the validity and accuracy of the method in dealing with the fluid-particle problem. Firstly, the different periodic channel lengths (1000, 2000 and 3000) are set in the flow direction to simulate the particle trajectory, and the results are shown in Figure 3 where we can see that the results are almost the same for the three lengths, so the channel length L and width H are selected as 2000×80∆x in the following simulation. The present numerical results of particle trajectory are shown in Figure 4 where the other results [28,29,30] are also given as a comparison, it can be seen that the results simulated by different methods agree well.

## 4. Results and Discussion

**Figure 5:**
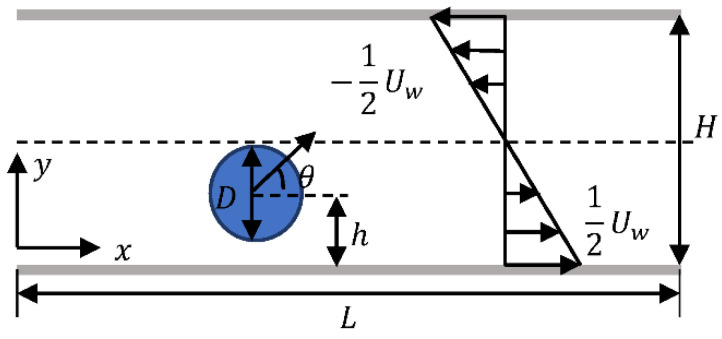
Initial condition of squirmer in a simple shear flow.

As shown in Figure 5, a squirmer with a diameter of 20∆x is released in a simple shear flow with an initial inclination angle θ and a distance h from the wall. The channel length L is set to 100D with *D* being the squirmer’s diameter, the blocking rate κ=D/H, and κ= 0.25 unless otherwise specified. The flow Reynolds number is defined as $Re_{p}=2U_{w}H/\mu$ with $U_{w}$ being the velocity difference between upper and lower walls. The swimming Reynolds number is defined as $Re_{s}=B_{1}d/2\nu$, where $B_{1}$ is related to swimming strength as shown in Equation (1) and ν is the kinematic viscosity. No-slip and impenetrable boundary conditions are used for the upper and lower walls, and periodic boundary conditions are used at the inlet and outlet.

### 4.1. Effect of Initial Condition on the Squirmer’s Motion

**Figure 6:**
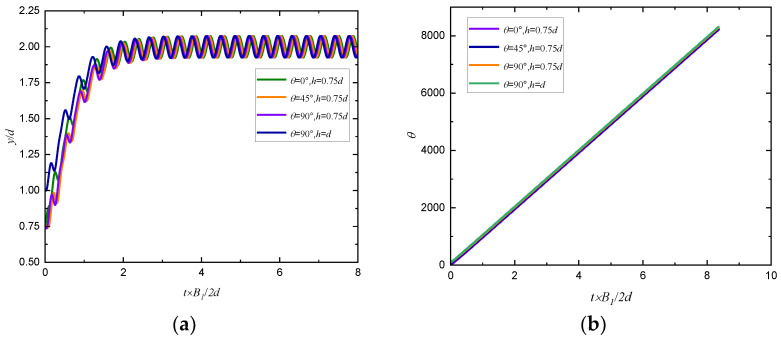
Changes of (**a**) trajectory and (**b**) orientation angle of a puller with time in simple shear flow (*Re_p_* = 80, *Re_s_* = 1, *β* = 5).

A puller with initial positions *h* = 0.75 *d*, *d*, 1.25 *d* and initial orientation angles θ=0°, 45°, 90° is released in a simple shear flow, and the changes of trajectory and orientation angle of puller with time are shown in Figure 6. We can see that the changes of trajectory and orientation angle of puller are independent of initial conditions. Therefore, the initial position and orientation angle are set to *h* = 0.75 *d* and *θ* = 0°, respectively, in the following simulation.

### 4.2. Effect of the Swimming Reynolds Number

**Figure 7:**
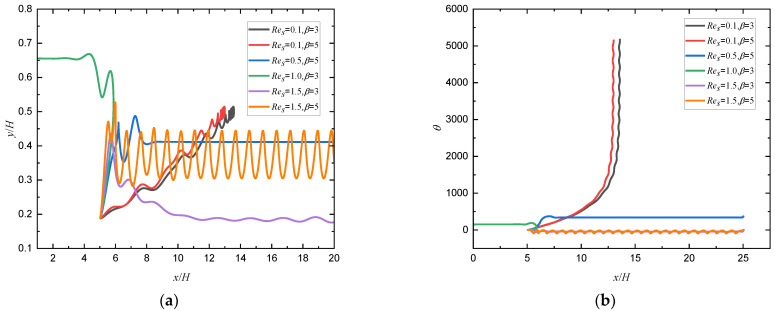
Changes of (**a**) trajectory and (**b**) orientation angle of a squirmer along the flow direction for different *Re_s_* and *β* (*Re_p_* = 80).

To explore the effect of $Re_{s}$ on the motion pattern of a squirmer swimming near the wall, a squirmer with a radius of 20∆x is released in the flow. Figure 7 shows the changes of trajectory and orientation angle of a squirmer along the flow direction for different *Re_s_* and *β*, it can be seen that there exist four distinct modes for squirmer motion, i.e., horizontal mode, attractive oscillation mode, oscillation mode, and chaotic mode. When $Re_{s}=0.1$, the squirmer will keep rolling as it moves to a constant position near the centerline of the channel, and will make a steady horizontal motion above ($Re_{s}=1.0,\;\beta =3$) or below ($Re_{s}=0.5,\;\beta =5$) the midline as $Re_{s}$ grows.

**Figure 8:**
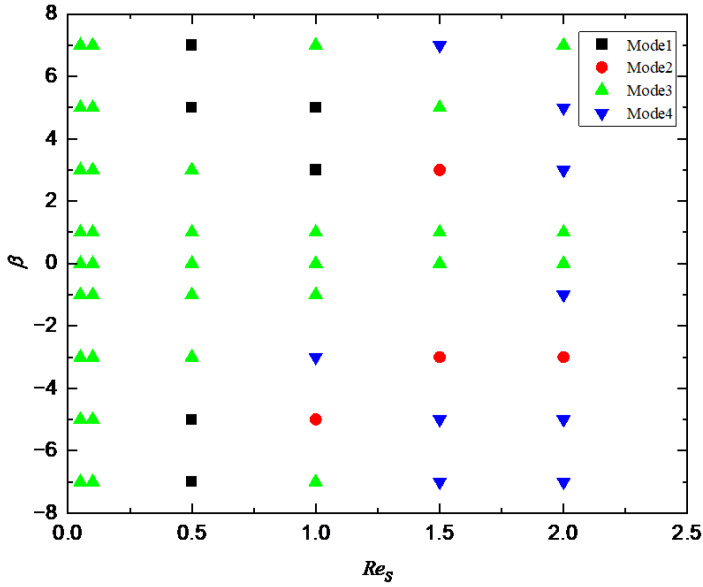
Phase diagram of *Re_s_* and *β* for the transition of different modes. Mode 1: horizontal; Mode 2: attractive oscillation; Mode 3: oscillation; Mode 4: chaos.

When $Re_{s}$ continues to increase to 1.5, the repulsion force exerted on the squirmer by the wall decreases because $Re_{s}=B_{1}d/2\nu$, the larger $\;Re_{s}$ is, the larger *d* is, the smaller the repulsion force is, as shown in Equation (3), and an attractive oscillation mode (β=5) or oscillation mode (β=3) will be formed near the wall, which is similar to the trend of squirmer moving near the non-slip wall [10]. When β increases from 3 to 5, the squirmer will escape from the wall and make a periodic stable motion at a specific distance from the wall at $Re_{s}=1.5$ because the self driving ability of squirmer is enhanced with the increase of swimming intensity. The phase diagram of *Re_s_* and *β* for the transition of different modes are shown in Figure 8. 

### 4.3. Effect of the Flow Reynolds Number

**Figure 9:**
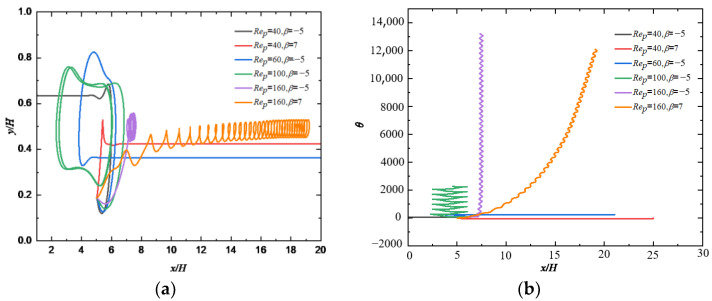
Changes of (**a**) trajectory and (**b**) orientation angle of a squirmer along the flow direction for different *Re_p_* and *β* (*Re_s_* = 0.5).

**Figure 10:**
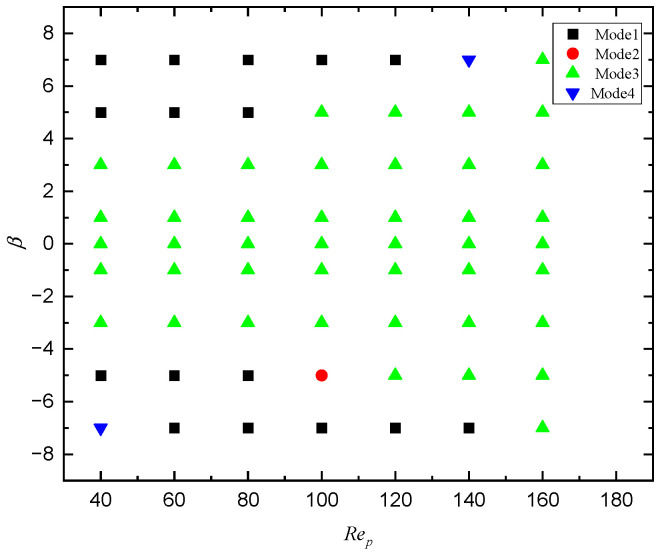
Phase diagram of *Re_p_* and *β* for the transition of different modes. Mode 1: horizontal; Mode 2: attractive oscillation; Mode 3: oscillation; Mode 4: chaos.

Effects of $Re_{p}$ and β on the motion pattern of a squirmer are shown in Figure 9 where we can see that the puller (β=7) will move towards the outlet of the flow and form a stable trajectory below the centerline when $Re_{p}=40$. However, the pusher (β=−5) will be attracted by the wall, move in the opposite direction after colliding with the wall first, and then move in the direction of the entrance across the center line, finally form a stable trajectory above the centerline. When $Re_{p}$ is increased to 60, the trajectory of the pusher (β=−5) is more complicated, but eventually a stable motion pattern is formed below the centerline. As $Re_{p}$ increases to 100, the trajectory of the pusher (β=−5) forms a closed loop. When $Re_{p}$ continues to increase to 160, the puller (β=7) will move to the centerline and form a spiral trajectory near the centerline. However, the pusher (β=−5) will keep rotating at a fixed position close to the centerline because an increase of $Re_{p}$ means an increase in shear strength, causing the pusher to gradually change from a stable motion state to a non-stop rotating or helical motion, which is similar to the sperm swimming up and down in the airflow when the airflow to the uterus is generated in the oviduct of mammals [11]. The phase diagram of *Re_p_* and *β* for the transition of different modes are shown in Figure 10. 

### 4.4. Effect of the Blocking Rate

**Figure 11:**
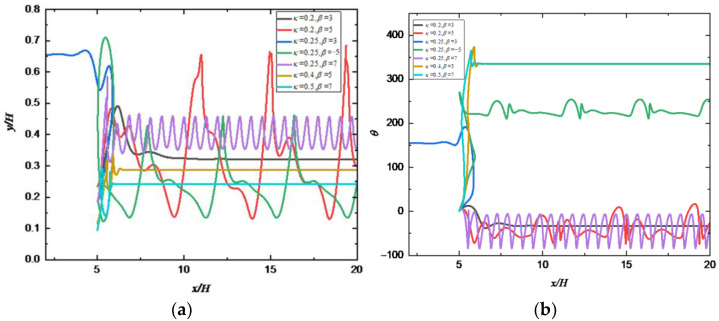
Changes of (**a**) trajectory and (**b**) orientation angle of a squirmer along the flow direction for different *κ* and *β* (*Re_s_* = 1.0, *Re_p_* = 80).

**Figure 12:**
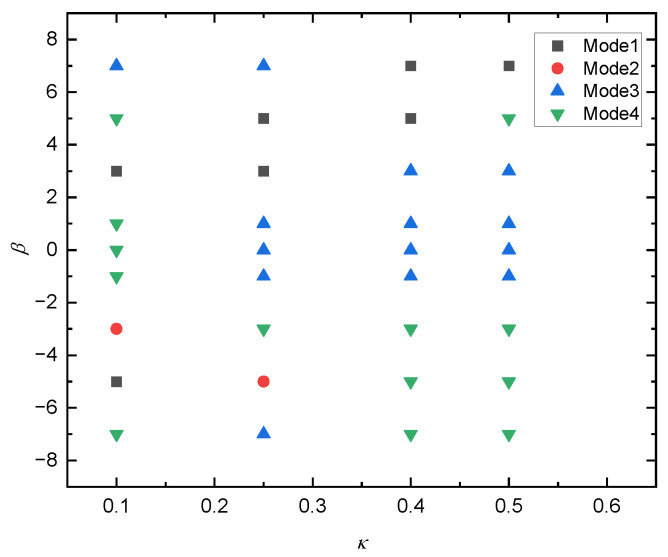
Phase diagram of κ and *β* for the transition of different modes. Mode 1: horizontal; Mode 2: attractive oscillation; Mode 3: oscillation; Mode 4: chaos.

Figure 11 shows the effects of *κ* and β on the motion pattern of a squirmer. It can be seen that, with the increase of *κ*, the motion pattern of a squirmer changes from both periodic motion (β=7) and attractive oscillation (β=5) to the horizontal motion, and the squirmer finally moves stably near the lower wall. The reason can be attributed to that increasing κ will change the magnitude of the total moment exerted on the squirmer, making it move horizontally and stably. As κ increases from 0.2 to 0.25, it can be clearly found that the motion direction of squirmer is diametrically opposite for β=3, the equilibrium position during stable motion also changes from below the centerline to above the centerline. Furthermore, with the increase of β, the motion pattern of squirmer changes from a horizontal mode (β=3) near the upper wall to an appealing oscillation mode (β=−5) near the lower wall at κ=0.25, and finally to an oscillation mode near the midline (β=7), the direction and strength of the attraction force of the wall to the squirmer will change. The phase diagram of κ and *β* for the transition of different modes are shown in Figure 12. 

## 5. Conclusions

The hydrodynamic properties of a squirmer type of self-propelled particle in a simple shear flow are investigated using the immersed boundary-lattice Boltzmann method. The present numerical results of particle trajectory are compared with the literature data, and the results agree well. The main conclusions are summarized as follows: there exist four distinct motion modes for the squirmer, i.e., horizontal mode, attractive oscillation mode, oscillation mode, and chaotic mode. The changes of trajectory and orientation angle of puller are independent of the initial conditions. Increasing *Re_s_* causes the motion mode to change from a constant tumbling near the centerline to a stable horizontal mode, even an oscillatory or appealing oscillatory mode near the wall. Increasing *β* will induce the squirmer to make periodic and stable motion at a specific distance from the wall. The squirmer will form a stable pattern of horizontal motion above or below the channel centerline when $Re_{p}=40-60$. At large $Re_{p}$, the squirmer’s trajectory will become closed loop, spiral, or even chaotic. Increasing *κ* will strengthen the wall’s attraction to the squirmer, make the squirmer’s motion progressively become steady, and cause the squirmer to move from an oscillation mode to attracting oscillation mode, and finally to horizontal motion mode. Increasing *β* will modify the strength and direction of the wall’s attraction to the squirmer. The conclusions obtained in this paper have reference value for mastering the hydrodynamic characteristics of self-propelled particles and controlling them. 

## Data Availability

Not applicable.
